# High-dose melphalan with autologous marrow for treatment of advanced neuroblastoma.

**DOI:** 10.1038/bjc.1982.11

**Published:** 1982-01

**Authors:** J. Pritchard, T. J. McElwain, J. Graham-Pole

## Abstract

**Images:**


*Br. J. Cancer* (1982) **45**, 86

### HIGH-DOSE MELPHALAN WITH AUTOLOGOUS MARROW FOR TREATMENT OF ADVANCED NEUROBLASTOMA

J. PRITCHARD*, T. J. McELWAIN†, AND J. GRAHAM-POLE‡

*From the *Department of Haematology and Oncology, Institute of Child Health and Hospital for Sick Children, Great Ormond Street, London WC1, †the Division of Medicine, Institute of Cancer Research and Royal Marsden Hospital, Sutton, Surrey and the ‡§Department of Medical Oncology, St. Bartholomew's Hospital, London EC1*

Received 13 January 1981 Accepted 9 October 1981

**Summary.—A group of 12 children with advanced neuroblastoma (7 Stage IV and 5 Stage III), selected by their initial response to chemotherapy with pulsed cyclophosphamide/vincristine/Adriamycin (CVA), were given consolidation therapy with high-dose melphalan (140 mg/m$^2$) and then surgical removal of residual disease. Twenty-two high-dose melphalan procedures were combined with autologous marrow grafting to offset myelotoxicity and were well tolerated. In each of 2 additional children, procedures carried out without marrow autografting led to serious marrow and mucosal toxicity. There were no treatment-related deaths. In 7/11 patients with evaluable computerized tomographic (CT) scans there was a decrease in maximum diameter of the primary tumour after melphalan. Complete response was achieved in 6 patients, of whom 3 are well and have no evidence of disease at 35, 33 and 18 months from completion of all treatment; however, although survival (median 23 months) of all 12 autografted patients is longer than that of 28 comparable children treated between 1970–77 with conventional chemotherapy (median 14 months) the difference is not statistically significant. High-dose melphalan is a safe and tolarable treatment in children when combined with autologous marrow grafting, but further study is required to determine whether the procedure can improve prognosis for patients with advanced neuroblastoma.**

ADVANCED NEUROBLASTOMA is one of the most lethal of all childhood neoplasms. The 2-year survival for children with Stage IV (Evans *et al.*, 1971) disease (excluding IVs patients) diagnosed and treated by us with conventional chemotherapy, radiotherapy and surgery is 10–15% (Ninane *et al.*, 1981); and this dismal experience is shared by others (Gasparini *et al.*, 1974; Finklestein *et al.*, 1979; Helson *et al.*, 1979). For stage III tumours, reported 2-year survival figures vary more, but in no series exceed 50%; our own experience is that Stage III patients with unresectable primary disease fare just as badly as those with Stage IV disease (Ninane *et al.*, 1981).

Between 1977 and 1979, in an attempt to improve the prognosis for children with advanced neuroblastoma, we studied the effect of high-dose melphalan (HDM) chemotherapy in 14 patients with Stage III or IV disease who had undergone "induction" chemotherapy with cyclophosphamide, vincristine and Adriamycin (CVA). We chose this approach because of previous experience of responses to HDM in patients with another neural-crest tumour, malignant melanoma (McElwain *et al.*, 1979*a*) and because its major side-

§ Current address: Dept. of Pediatric Hematology/Oncology, Rainbow Babies and Children's Hospital, Case Western Reserve University, Cleveland, Ohio 44106, U.S.A.

effect, myelotoxicity, could be overcome by autologous marrow grafting (McElwain *et al.*, 1979*b*). The short plasma half-life of melphalan permits the return of anti-coagulated, non-cryopreserved marrow to the patient 8 h after harvesting (McElwain *et al.*, 1979*b*) thus eliminating the technical problems of long-term marrow storage (Abrams *et al.*, 1980). A prerequisite for marrow autografting, carried our in 12 patients, was that the child's marrow was morphologically clear of tumour cells at the time of harvesting. Two patients, whose marrows still contained tumour cells despite CVA therapy, were also given HDM, but without marrow support.

In this paper, we assess the practicability and value of this treatment approach in 14 patients with advanced neuroblastoma. Some of our results have already been published in preliminary form (McElwain *et al.*, 1979*a*).

### PATIENTS AND METHODS

*Diagnosis and staging.*—In each of the patients (Table I), a diagnosis of neuroblastoma was established by either (a) tissue biopsy or (b) significantly elevated 24h urinary vanillyl-mandelic acid (VMA) excretion and cytological evidence of marrow infiltration by tumour cells. Surgery was eventually performed in 10 patients and yielded histological confirmation in 9 of them. Staging investigations included full blood count, chest X-rays, IVU, skeletal survey, liver and bone radioisotope scans, and marrow aspirates and/or trephine biopsies from at least 2 sites. Computerized tomography (CT) and ultrasound examinations were used serially to assess the size of the primary tumours. In 5 patients, lymph nodes were involved; however, because most of the primary tumours were abdominal and since

FIG. 1.—Treatment schema: Some patients (see Table I) received more than 6 courses of cyclophosphamide/vincristine/adriamycin (CVA). Not all patients underwent surgery and radiotherapy (see text). * mg/M2; MAG = autologous bone marrow graft; S = surgery; B = biopsy; RT = radiotherapy.

TABLE I.—*Clinical details of* 14 *patients at presentation, details of induction chemotherapy ("CVA"—see Fig.* 1) *and marrow status prior to HDM*

| Patient | Age at diagnosis (years) | Stage | Disease sites at presentation* — Site of primary§ | Lymph nodes | Liver | Bone | Marrow | Other | No. of courses of CVA | Marrow status after CVA |
|---|---|---|---|---|---|---|---|---|---|---|
| 1 | 2 | III | T/A | − | − | − | − | Pleura | 12 | − |
| 2 | 4 | IV | A | − | − | + | + | Dura | 6 | − |
| 3 | 3 | IV | A | + | − | − | − | − | 12 | − |
| 4 | 16 | IV | ? | − | + | − | − | − | 8 | − |
| 5 | 4 | IV | A | + | − | + | + | Scalp | 7 | − |
| 6 | 2 | IV | T/A | + | − | + | + | − | 8 | − |
| 7 | 9 | IV | A | + | − | − | + | Pleura | 6† | − |
| 8 | 13 | III | T/A | − | − | − | − | Pleura | 6‡ | − |
| 9 | 2 | III | A | − | − | − | − | − | 9 | − |
| 10 | 2 | III | A | − | − | − | − | − | 6 | − |
| 11 | 5 | IV | A | − | ± | + | + | − | 7 | − |
| 12 | 8/12 | III | A | − | − | − | − | − | 8 | − |
| 13 | 5 | IV | A | − | + | + | + | Dura? | 6 | + |
| 14 | 9 | IV | A | + | − | + | + | − | 6 | + |

? = primary unknown.
* { + = involved by tumour. − = no measurable involvement by tumour.
† = patient had 6 additional pulses of cyclophosphamide during induction treatment.
‡ = patient had induction therapy with Adriamycin alone.
§ A = abdominal; T = thorax. Pleural involvement in 2 cases was direct extension of primary but in the 3rd was judged to be metastatic.

the rate of initial laparotomy was low, this is almost certainly an underestimate. All but patient 9 excreted abnormally high levels of urinary VMA.

*Treatment schema.*—This is shown in Fig. 1. To be considered for autologous marrow grafting, we required that patients' marrow aspirates and/or trephine biopsies, from at least 2 sites, were morphologically clear of neuroblastoma. Of the 12 eligible children, 11 had received 6–12 (median 8) courses of CVA (Table I) prior to HDM, whilst a single patient with Stage III disease had received only Adriamycin, but was considered eligible for the study. Patients 13 and 14, whose marrows showed persistent infiltration by tumour after 6 courses of CVA, were ineligible for autografting.

*Procedure for high-dose melphalan (HDM).*—Patients for HDM were completely re-staged using the investigations listed above. Full blood counts and renal function were confirmed as normal. Oral non-absorbable antibiotics ('FRACON', [Storring *et al.*, 1977] or amphotericin/neomycin) were prescribed. Under general anaesthesia, a bladder catheter and a central venous line or Hickman catheter were inserted just before heparinization of the patient, and marrow was harvested as previously described for adults (McElwain *et al.*, 1979*a*; Thomas *et al.*, 1975). One–2 ml marrow aspirates were taken into 20ml heparinized syringes from multiple sites in both anterior and posterior iliac crests and, in one case, the tibiae. Median yields of nucleated marrow cells were, for the first autograft, $4{\cdot}1 \times 10^8$/kg (range $1{\cdot}8$–$33{\cdot}0 \times 10^8$) and, for the second, $4{\cdot}6 \times 10^8$/kg (range $2{\cdot}2$–$7{\cdot}7 \times 10^8$/kg). Whole blood, equal in volume to that of the marrow aspirate, was transfused during the procedure. No protamine sulphate was given. The marrow was stored, at 4°C, in a plastic transfer pack. On return to the ward, patients were nursed in cubicles using a simple "reverse isolation" technique with hand-washing and gowning. One parent "lived in" with each child and carried out many of the simpler nursing procedures. After recovery from anaesthesia, a few patients required a single dose of morphine for discomfort from sites of marrow aspiration. Melphalan (140 mg/i.v. as bolus) was given through the central venous line 1–2 h after recovery from anaesthesia; a brisk diuresis (6–8 ml urine/kg/h) was then induced using frusemide (2 mg/kg) and normal saline with added potassium. Eight hours after melphalan the marrow was returned via the central venous line. Twenty-four hours after melphalan, the bladder catheter was removed and the diuresis discontinued, but most children required i.v. fluids for a further 24–48 h because of nausea and anorexia.

Appropriate supportive care was given during the 2–3 weeks of pancytopenia following HDM. After 4–6 weeks at home and recovery of their blood count, 10 patients returned to hospital for a second HDM procedure. Patient 8 declined further chemotherapy and abnormal liver function tests post-HDM were considered a contra-indication to a second procedure in patient 4.

*Further treatment and follow-up.*—No further chemotherapy was given, but, 6–10 weeks after HDM, 9 patients underwent exploratory leparotomy in an attempt to remove residual disease or, at least, obtain samples for histology. Radiotherapy was given to 2 patients with localized residual abdominal disease after surgery: patient 9 received a dose (25 Gy in 10 fractions over 29 days) considered technically and therapeutically adequate, but in patient 10 a final dose of 15 Gy (considered suboptimal) was achieved only after several interruptions because of thrombocytopenia.

All treatment was concluded between 9 and 15 months (median 12) from diagnosis. Subsequent follow-up was with regular clinical examination and VMA estimations, 4-monthly marrow examinations and abdominal ultrasound and bone scans or skeletal surveys when indicated.

### RESULTS OF TREATMENT

### *Tumour response (Table II)*

Of the 12 patients who, just before HDM, had localized disease only, 11 had evaluable serial CT scans, and 7 showed some shrinkage of maximum tumour diameter as a result of the treatment (*e.g.* Fig. 2) "Complete response" (CR) status (defined as: no clinical or imaging evidence of primary or secondary tumour, normal marrow aspirates and/or trephines and normal urinary VMA) was achieved in 6 of these 12 patients, but in patients 1, 2, 5 and 11, only after surgical excision of residual disease. Six children never

TABLE II.—*Disease response to HDM and to all treatment, sites of relapse and outcome*

| Patient | Measurable disease prior to HDM | Measurable disease after HDM | Measurable disease after all $R_x$ | Site of relapse | Outcome: Status | Outcome: Months from initial $R_x$ |
|---|---|---|---|---|---|---|
| 1 | P | P↓ | CR | — | NED | 45 |
| 2 | P | P | CR | — | NED | 43 |
| 3 | P | P | P | P, LN | Died | 18 |
| 4 | LN, VMA | VMA | VMA | H, BM | Died | 31 |
| 5 | P, VMA | P↓ | CR?* | BM | Died | 13 |
| 6 | P | P | P | P, BM | Died | 32 |
| 7 | CR | CR | CR | pleura, BM | Died | 23 |
| 8 | P | P↓ | P | P | Died | 41 |
| 9 | P | P↓ | P↓ | H | Died | 11 |
| 10 | P | P↓ | P↓ | P, lung | Died | 20 |
| 11 | P, ?LN | P↓ | CR | B, BM | Died | 17 |
| 12 | P, VMA | CR (P↓) | CR | — | NED | 26 |
| 13 | P, H, B, BM, VMA | P, H, B, BM* | P, H, B, BM, VMA | Progressive disease | Died | 12 |
| 14 | P, BM, VMA | P, BM, VMA | P, BM, VMA | Progressive disease | Died | 29 |

Abbreviations: P = primary tumour

↓ = some decrease in maximum diameter of P via HDM surgery

LN = lymph nodes

H = liver

NED = no evidence of tumour

VMA = increased urinary VMA excretion

B = bone

BM = marrow

* = VMA not estimated

FIG. 2.—CT scans of the abdomen demonstrating left adrenal primary tumour of patient 10 immediately before (a) and after (b) 2 courses of HDM. Over 50% shrinkage of maximum diameter and increased calcification are shown.

achieved CR. Patients 3 and 8 had residual unresectable gross disease, whilst in patients 9 and 10 microscopic disease remained after "second-look surgery" and was treated with radiotherapy. In patient 6 a residual primary para-spinal tumour, detectable by CT scanning, could not be located at laparotomy, whilst, for patient 4, persistently high urinary VMA excretion was the only evidence of residual disease. Neither child (patients 13 and 14) with metastatic disease at the time of HDM showed objective response to this treatment.

FIG. 3.—Progress chart for patient 10. During induction blood counts were only performed just before each pulse of CVA. Chemotherapy doses are in mg/M², L-PAM = melphalan, ↑ = elevated urinary VMA excretion, sl ↑ = slightly elevated VMA excretion, BT = red cell transfusion, ✝ = child died.

FIG. 4.—Survival after initial response to treatment; comparison of 12 HDM/autograft patients [— — —] (this series) with 28 patients [———] (Ninane *et al.*, 1981) with similar initial response to CVA or CV who continued treatment with this chemotherapy, ± radiotherapy ± surgery to local disease. ○ denotes disease-free survivor.

*Survival and relapse* (*Fig.* 2, *Table II*)

Of the 12 children receiving autografts 3 (patients 1, 2 and 12) remain alive, without evidence of neuroblastoma, 35, 33 and 18 months from the end of all treatment. All 3 had achieved CR, one after chemotherapy alone and 2 after chemotherapy and surgery. The 9 other children have died of disseminated tumour recurrence; in 3 there was simultaneous recurrent disease at the primary site. Both non-autografted children experienced relief of symptoms after HDM; one survived for 8 months and the other, with the help of alternative chemotherapy, for 22 months before dying of metastatic tumour.

*Complications*

A total of 24 HDM procedures were carried out in 14 patients, 22 with autologous marrow support. There were no complications from heparin anticoagulation even without protamine reversal. Administration of melphalan through a central venous line eliminated the severe local discomfort of administration by peripheral vein. No hypotensive episodes, previously noted by us in adults (McElwain *et al.*, 1979*a*) were seen in these children, possibly because careful volume-for-volume whole-blood replacement of aspirated marrow was carried out during the procedure. Recovery of total leucocyte counts to $1\cdot0 \times 10^9$/l, of neutrophil counts to $0\cdot8 \times 10^9$/l and of platelet counts to $80 \times 10^9$/l occurred at medians of 9·5 (range 3–21), 16 (9–28) and 27·5 (9–36) days respectively after the first HDM treatment, and at medians of 12 (6–20), 25·5 (11–36) and 37·5 (28-indefinite—see Discussion) days after the second. These figures are comparable to those previously reported (McElwain *et al.*, 1979*a*, *b*) for adults after HDM.

All children received platelet support at counts below $20 \times 10^9$/l; only one instance of overt bleeding was detected, and responded promptly to further platelet transfusion. After 2 courses of HDM, 3 patients, each of whom ultimately succumbed to tumour, had persistent thrombocytopenia stabilizing at $50–100 \times 10^9$/l (see Fig. 4).

Since total nucleated cell yields and marrow volumes were similar for both harvests in the 10 patients who underwent 2 autografts, this incomplete haemopoietic reconstitution was not apparently related to a reduced marrow "dose" at the second procedure. The 2 patients without autografts showed slower blood-count recovery than the grafted patients; consequently, they spent longer in hospital (median 22·5 days for grafted and 30 days for non-grafted patients) and experienced the only 2 serious infective complications in the entire series. One child suffered severe stomatitis, with secondary thrush and bleeding, and severe diarrhoea; the second developed an *E. coli* septicaemia, the only documented systemic infection in the entire group. Both patients recovered, but only after receiving white-cell transfusions as well as antibiotics. All other patients received empirical broad-spectrum antibiotic therapy after development of pyrexia of unknown origin whilst neutropenic, but no bacterial pathogens were isolated. No graft-versus-host disease was recognized.

Serum IgG, IgM and IgA were normal throughout treatment of 2 patients, but there was a fall in lymphocyte response to PHA and loss of skin reactivity to candidin just after HDM. Unfortunately, neither patient survived long enough to permit retesting at a longer interval after melphalan. Serious viral or fungal infections, however, did not occur; one child developed shingles and another measles 22 months after their last chemotherapy but recovered without complications.

Other complications of treatment were rare. One patient developed hyperuricaemia (1·1 mM) 3 days after his first HDM treatment; since this episode coincided with a rapid decrease in size of the primary tumour, it was presumed to be the consequence of cell lysis, a phenomenon we have never encountered in children with neuroblastoma receiving conventional chemotherapy. Serial electrolyte estimations after HDM were normal, as were liver-function tests, except in one child whose SGOT and SGPT were moderately raised, without jaundice, for 6–8 weeks. Most children lost $> 10\%$ of their body weight as a result of each HDM treatment but quickly regained it after discharge from hospital and before a second procedure.

### DISCUSSION

In designing this protocol we had in mind the characteristics of relapse in patients with Stage III and IV neuroblastoma. After partial response to conventional treatment, "recurrence" is generally local within a median time of about 6 months, whilst after CR tumour recurrence characteristically occurs at metastatic sites after a median of 15 months (Ninane *et al.*, 1981). Surgical resection of disease after conventional chemotherapy, with or without radiotherapy, does not appear to improve survival (Finklestein *et al.*, 1979). Sequential segmental whole-body irradiation did not improve duration of first response in one reported series (Green *et al.*, 1976) and although single-dose 10 Gy total-body irradiation followed by allogeneic marrow grafting from an HL-A compatible sibling requires further investigation, early results are not encouraging (Klemperer *et al.*, 1976; Evans & d'Angio, personal communication; McElwain *et al.*, personal communication). We therefore decided to explore the possibility that high-dose chemotherapy, by eliminating metastases and shrinking primary disease, might lead to improved CR rate and outcome. Of drugs conventionally active against neuroblastoma, only some alkylating agents (Thurman *et al.*, 1964) lack predictable dose-related specific organ toxicity. We decided against the use of cyclophosphamide because of (a) the risk of cystitis (Phillips *et al.*, 1961) and cardiomyopathy (Buchner *et al.*, 1972), (b) the need, after CVA induction therapy, to expose the tumours to a "new" agent in order to side-step the problem of emerging drug resistance and (c) the reported failure of high-dose cyclophosphamide to improve the prognosis in advanced neuroblastoma (Helson, 1979; Nitschke *et al.*, 1979*a*). Encouraged by response rates to Peptichemio (de Bernardi *et al.*, 1978) up to 92%, we decided to use the closely related alkylating agent melphalan for high-dose consolidation. The only previous use of melphalan in neuroblastoma was not encouraging, but in that study (Fernbach *et al.*, 1968) and in contrast to our own plans, a low-dose schedule of oral administration was used. Our protocol was so designed that HDM would be introduced at a time of estimated minimum tumour burden; we measured its effect by serial CT scanning, laparotomy after treatment and crude survival.

Marrow harvesting, even in the smallest children, presented no particular problems, though in one 2-year-old tibial marrow was aspirated to reach an adequate cell dose. Of considerable importance was the fact that none of these children had previously received pelvic irradiation, which greatly reduces the chance of an adequate cell yield. The mean dose was similar to that used in adults (McElwain *et al.*, 1979*a*). HDM was well tolerated in all patients who received autologous marrow grafts. No life-threatening episodes, apart from one septicaemia, were encountered in the entire series, but our experience of serious complications with the 2 non-autografted children, their marrows admittedly compromised by tumour infiltration, together with our previous findings in adults (McElwain *et al.*, 1979*b*) convince us that marrow autografting significantly reduces the morbidity from HDM therapy. Oral non-absorbable antibiotics and nystatin were administered, but compliance was poor because of mucositis and nausea. We now use the more palatable combination of Cotrimoxazole and nystatin for patients undergoing HDM therapy. Energetic diuresis was used after melphalan, because 2 adult patients, early in our experience (McElwain *et al.*, 1979*a*) developed compromised renal function post-HDM. We now have evidence that diuresis is unnecessary. Weight loss of $>10\%$ in each of our patients might have been prevented by early i.v. feeding: we now invariably start parenteral feeding immediately after HDM.

The 14 children reported here represent less than two-thirds of the 22 patients referred to our 3 hospitals with a diagnosis of Stage III or IV neuroblastoma during the 2 years of the study. Of the remainder, 6 were considered ineligible for HDM because of non-response to initial (CVA) chemotherapy, 1 because of pre-existing brain damage, and the last because of a chemotherapy-induced malabsorption syndrome. That our patients are a "selected" group thus primarily reflects the failure of CVA as an induction regimen.

Three children (Stage III patients 1 and 12, Stage IV patient 2) are well and free of evidence of disease between $1\frac{1}{2}$–3 years from the end of treatment. The survival of the 12 patients who had localized disease just before HDM (median 23 months) is, however, not significantly different (Logrank test $P = 0{\cdot}76$) from that of previous patients achieving clinical CR by conventional therapy (median 14 months—Ninane *et al.*, 1981). Moreover, neither child with overt metastases at the time of HDM showed objective response to the treatment. However, since all therapy was completed 9–15 months after diagnosis, the quality of the subsequent lives of children receiving HDM was substantially better than those treated prior to 1977.

In 8/9 relapsing patients, disease returned at metastatic sites; however, there was simultaneous local recurrence in 3 of these children, each of whom had had incomplete surgical resection, and one had a suboptimal radiation dose as well. We therefore feel that a more aggressive approach towards residual localized disease, using both surgery and high-dose age-adjusted radiation therapy, even in the face of a falling blood count, would have been justified. There are several possible explanations for relapse at *distant* sites. The most likely is that HDM failed to destroy occult metastases; alternatively, the "new" metastases might represent re-seeding of tumour cells either present in the reinfused autologous marrow or from the primary site. The pattern and timing of reappearance of metastases does not permit distinction between these possibilities, of which the third seems unlikely in the 5 cases in which secondary disease recurred without evidence of local relapse.

We conclude that HDM is a safe and tolerable procedure in children when combined with autologous marrow grafting, expert nursing and close parental support in specialized units. Because of encouraging results of high-dose chemotherapy, including melphalan, in other forms of cancer (Ziegler *et al.*, 1977, Cornbleet *et al.*, 1981) and despite the disappointing overall results in this series of children, we continue to use HDM consolidation in patients with advanced neuroblastoma. We now use more intensive induction therapy, including *cis*-Platinum and VM-26, agents which have been shown to be active both individually (Rivera *et al.*, 1977, Nitschke *et al.*, 1979*b*) and together (Hayes *et al.*, 1981) in neuroblastoma, and pay particular attention to the radiation and surgical therapy of residual primary disease. Finally, we are exploring the possibility that monoclonal antibodies, known to bind to human neuroblastoma cells (Kemshead *et al.*, 1981) might be used to "clean up" autologous marrow before reinfusion.

The authors gratefully acknowledge the high standard of care offered to these patients and their families by the nursing and medical staff of Princess Chula Ward, Royal Marsden Hospital, Ward 3AB, Hospital for Sick Children and Kenton Ward, St Bartholomew's Hospital. We also thank Drs Ruth Sandland, Ann Barrett, David Lawson and Professor J. S. Malpas for many helpful discussions, the paediatricians who referred patients to our care, and the haematologists who were responsible for obtaining marrow for autografting. Mr Herbert Eckstein and Mr James Dickson performed the surgical procedures. Dr Janet Husband, Dr J. S. MacDonald and their staff carried out and interpreted the CT scans in the Cancer Research Campaign's CT Scanning Unit, Royal Marsden Hospital. The assistance of Drs Ian Hann, David Hedley and Jacques Ninane during the study and the helpful comments of Dr Judith Chessells and Professor R. M. Hardisty on the manuscript were much appreciated. Dr Pritchard is supported by a grant from the Leukaemia Research Fund.

### REFERENCES

ABRAMS, R. A., GLAUBIGER, D., SIMON, R., LICHTER, A. & DEISSEROTH, A. B. (1980) Haemopoietic recovery in Ewing's sarcoma after intensive combination therapy and autologous marrow infusion. *Lancet*, ii, 385.

BUCHNER, C. O., RUDOLPH, R. H. & FEFER, E. L. (1972) High-dose cyclophosphamide for malignant disease: Toxicity, tumour response and the effects of stored autologous marrow. *Cancer*, **29**, 257.

CORNBLEET, M. A., CORRINGHAM, R. E. T., PRENTICE, H. G., BOESEN, E. M. & MCELWAIN, T. (1981) Treatment of Ewing's sarcoma with high-dose melphalan. *Cancer Treat. Rep.*, **65**, 241.

DE BERNARDI, B., COMELLI, C., COZZUTTO, C., LAMEDICA, G., MORI, P. G. & MASSIMO, L. (1978) Peptichemio in advanced neuroblastoma. *Cancer Treat. Rep.*, **62**, 811.

EVANS, A. E., D'ANGIO, G. J. & RANDOLPH, J. (1971) A proposed staging for children with neuroblastoma. *Cancer*, **27**, 374.

FERNBACH, D. J., HADDY, T. B., HOLCOMB, T. M., STUCHY, W. J. JR., SULLIVAN, M. P. & WATKINS, W. L. (1968) L-sarcolysin (NSC 8806) therapy for children with metastatic neuroblastoma. *Cancer Chemother. Rep.*, **52**, 293.

FINKLESTEIN, J. Z., KLEMPERER, M. R., EVANS, A. E. & 8 others (1979) Multiagent chemotherapy for children with metastatic neuroblastoma. A report from the Children's Cancer Study Group. *Med. Pediat. Oncol.*, **6**, 179.

GASPARINI, M., BELLANI, F. F., MUSUMECI, R. & BONADONNA, G. (1974) Response and survival of patients with metastatic neuroblastoma after combination chemotherapy with adriamycin

(NSC-123127), cyclophosphamide (NSC-26271) and vincristine (NSC-67574). *Cancer Chemother. Rep.*, **58**, 365.

GREEN, A. A., HUSTU, H. O., PALMER, R. & PINKEL, D. (1976) Total-body sequential segmental irradiation and combination chemotherapy for children with disseminated neuroblastoma. *Cancer*, **38**, 2250.

HAYES, F. A., GREEN, A. A., CASPER, J., COMET, J. & EVANS, W. E. (1981) Clinical evaluation of sequentially scheduled Cis-platin and VM-26 in neuroblastoma: response and toxicity. *Cancer*, (In press).

HELSON, L. (1979) Investigational chemotherapy of neuroblastoma. *J. Florida Med. Assoc.*, **66**, 284.

KEMSHEAD, J. T., WALSH, F., PRITCHARD, J. & GREAVES, M. (1981) Monoclonal antibody to ganglioside GQ discriminates between haemopoietic cells and infiltrating neuroblastoma tumour cells in bone marrow. *Int. J. Cancer*, **27**, 447.

KLEMPERER, M. R., GARRICK, D., SHIGOEKA, A., LEE, H. & SIEGEL, S. (1976) Attempted treatment fo a child with metastatic neuroblastoma employing syngeneic marrow transplantation. *Transplantation*, **21**, 161.

McELWAIN, T. J., HEDLEY, D. W., BURTON, G. & 10 others (1979*a*) Marrow autotransplantation accelerates haematological recovery in patients with melanoma treated with high-dose melphalan. *Br. J. Cancer*, **40**, 72.

McELWAIN, T. J., HEDLEY, D. W., GORDON, M. Y., JARMAN, M. & PRITCHARD, J. (1979*b*) High-dose melphalan and non-cryopreserved autologous bone marrow treatment of malignant melanoma and neuroblastoma. *Exp. Haematol.*, **7**, (Suppl. 5) 360.

NINANE, J., PRITCHARD, J. & MALPAS, J. S. (1981) Treatment of advanced neuroblastoma: Does adriamycin contribute? *Arch. Dis. Child.* **56**, 544.

NITSCHKE, R., CANGIR, A., CRIST, W. & BERRY, D. H. (1980) Intensive chemotherapy for metastatic neuroblastoma. *Med. Pediat. Oncol.*, **8**, 281.

NITSCHKE, R., FAGUNDO, R., BERRY, D. H. & FALLETTA, J. M. (1979) Weekly administration of cis-dichloro-diammine platinum (II) in solid childhood tumours; a Southwestern Oncology Group Study. *Cancer Treat. Rep.*, **63**, 497.

PHILLIPS, F. S., STERNBERG, S. S., CRONIN, A. P. & VIDAL, P. M. (1961) Cyclophosphamide and urinary bladder toxicity. *Cancer Res.*, **21**, 1577.

RIVERA, G., GREEN, A. A., HAYES, A., AVERY, T. & PRATT, C. (1977) Epipodophyllotoxin VM-26 in the treatment of childhood neuroblastoma. *Cancer Treat. Rep.*, **61**, 1243.

STORRING, R. A., JAMESON, B., McELWAIN, T. J., WILTSHAW, E., SPIERS, A. S. D. & GAYA, H. (1977) Oral non-absorbed antibiotics prevent infection in acute lymphoblastic leukaemia. *Lancet*, ii, 837.

THOMAS, E. D., STORB, R., CLIFT, R. A. & 6 others. (1975). Bone marrow transplantation. *N. Engl. J. Med.*, **292**, 832; 895.

THURMAN, W. G., FERNBACH, D. J. & SULLIVAN, M. P. (1964) Cyclophosphamide therapy in childhood neuroblastoma. *N. Engl. J. Med.*, **270**, 1336.

ZIEGLER, J. L., DEISSEROTH, A. B., APPLEBAUM, F. R. & GRAW, R. G. JR. (1977) Burkitt's lymphoma: A model for intensive chemotherapy. *Semin. Oncol.*, **4**, 317.


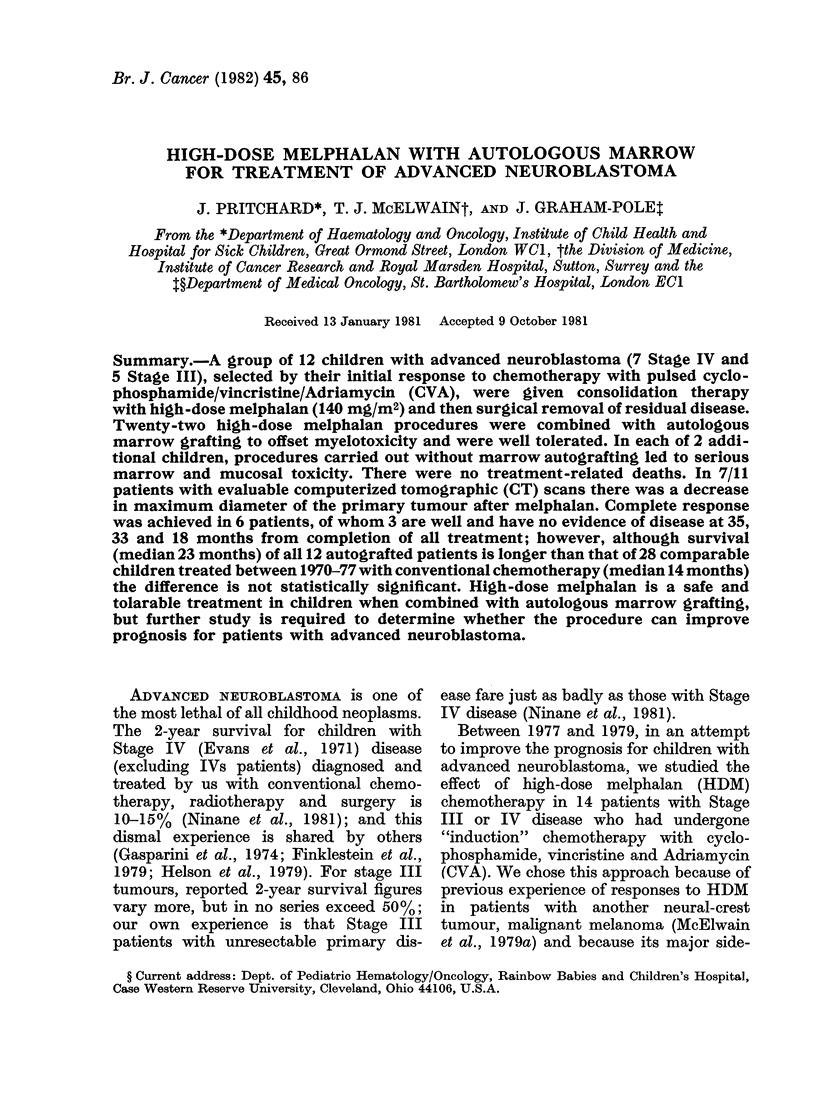

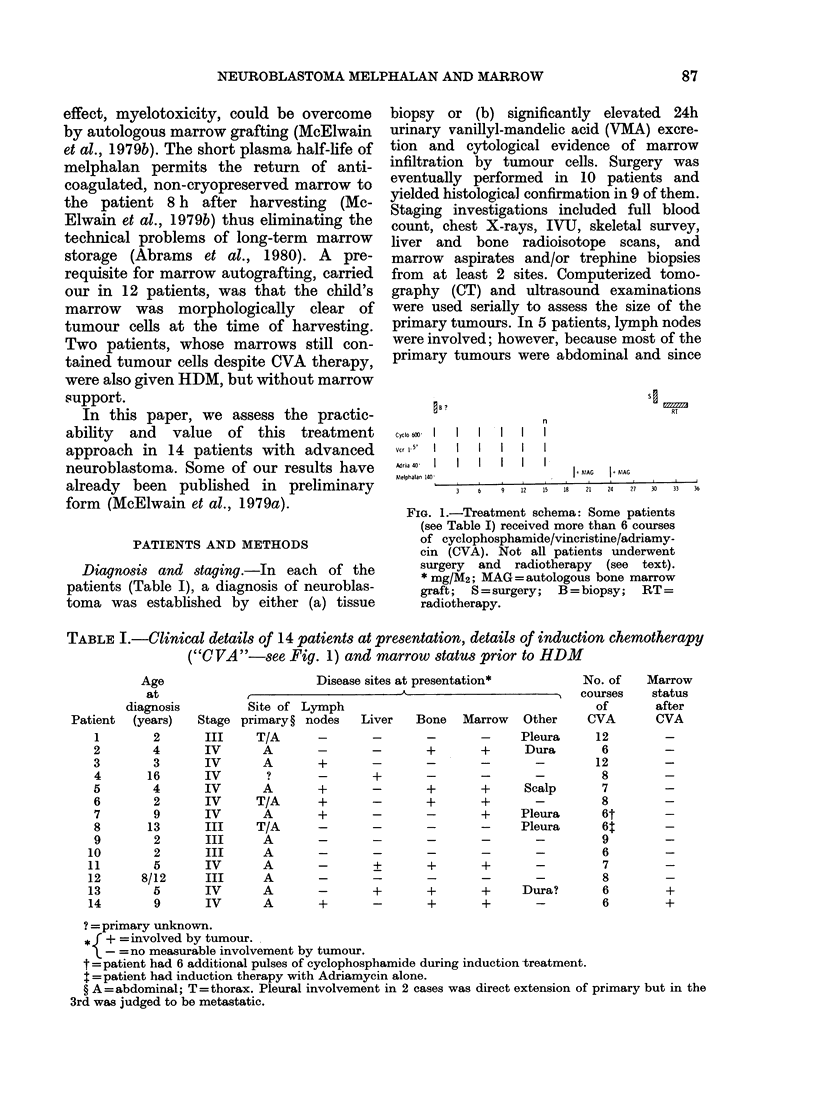

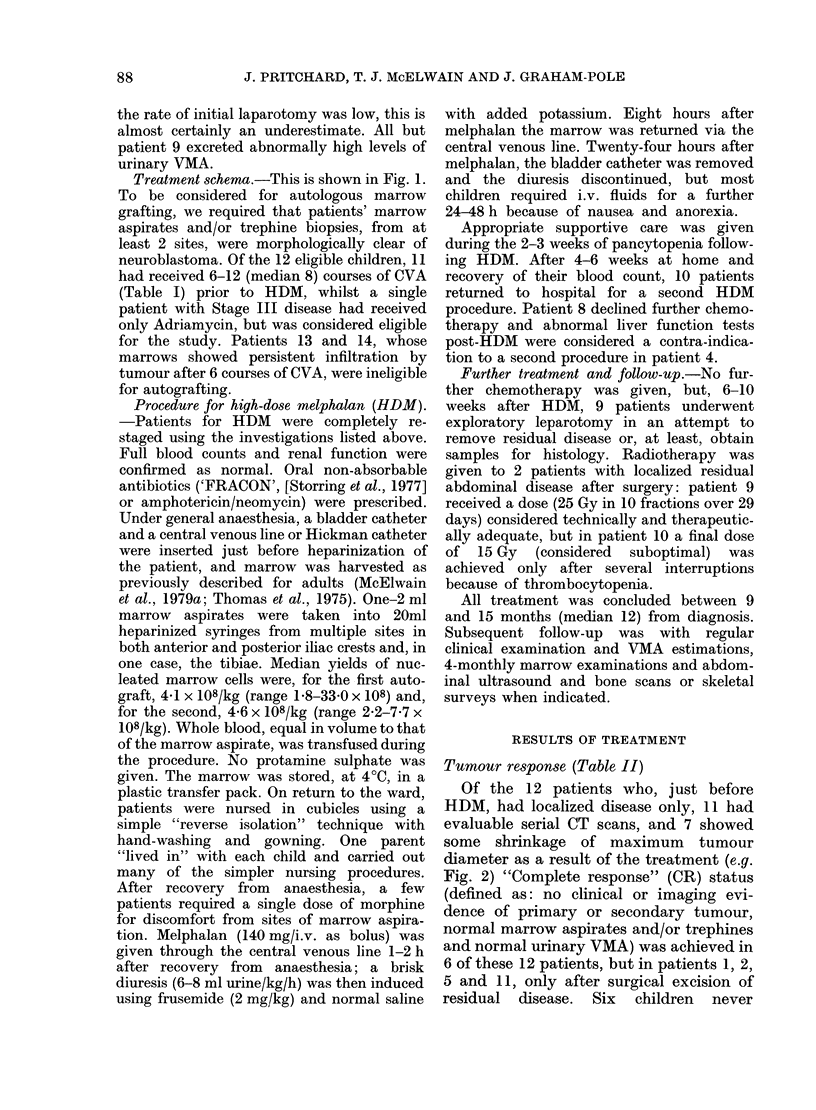

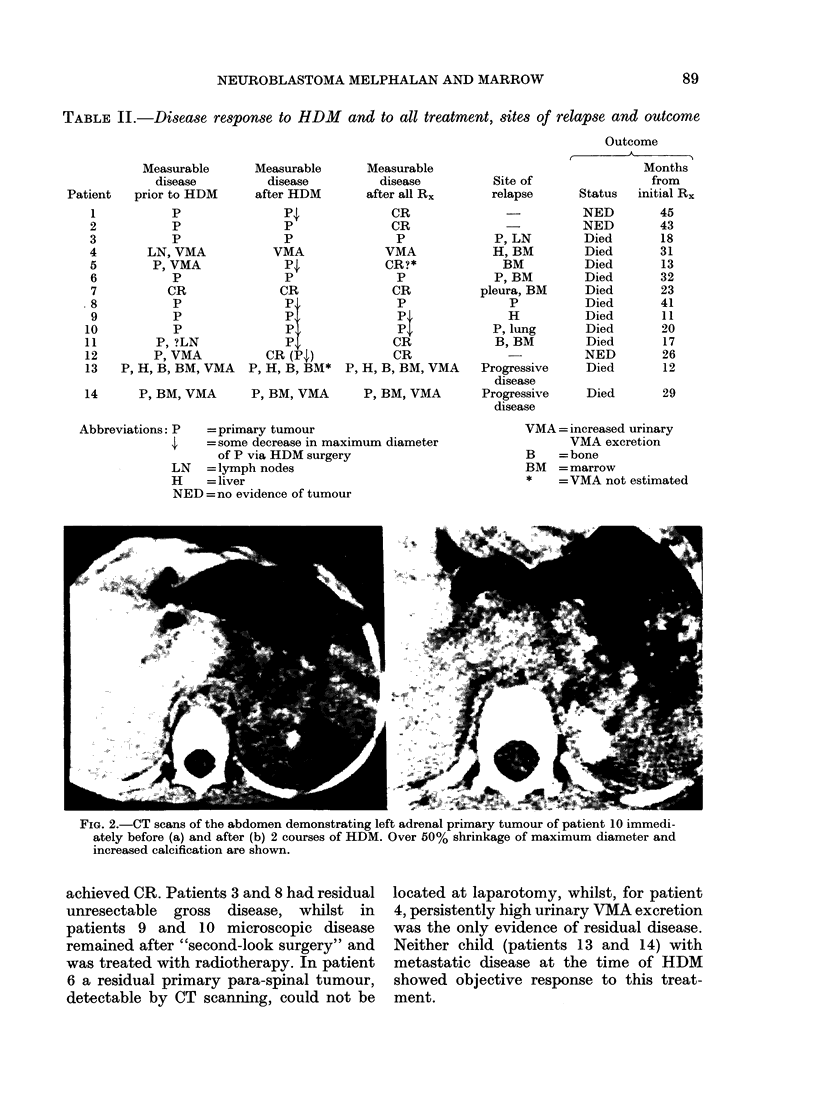

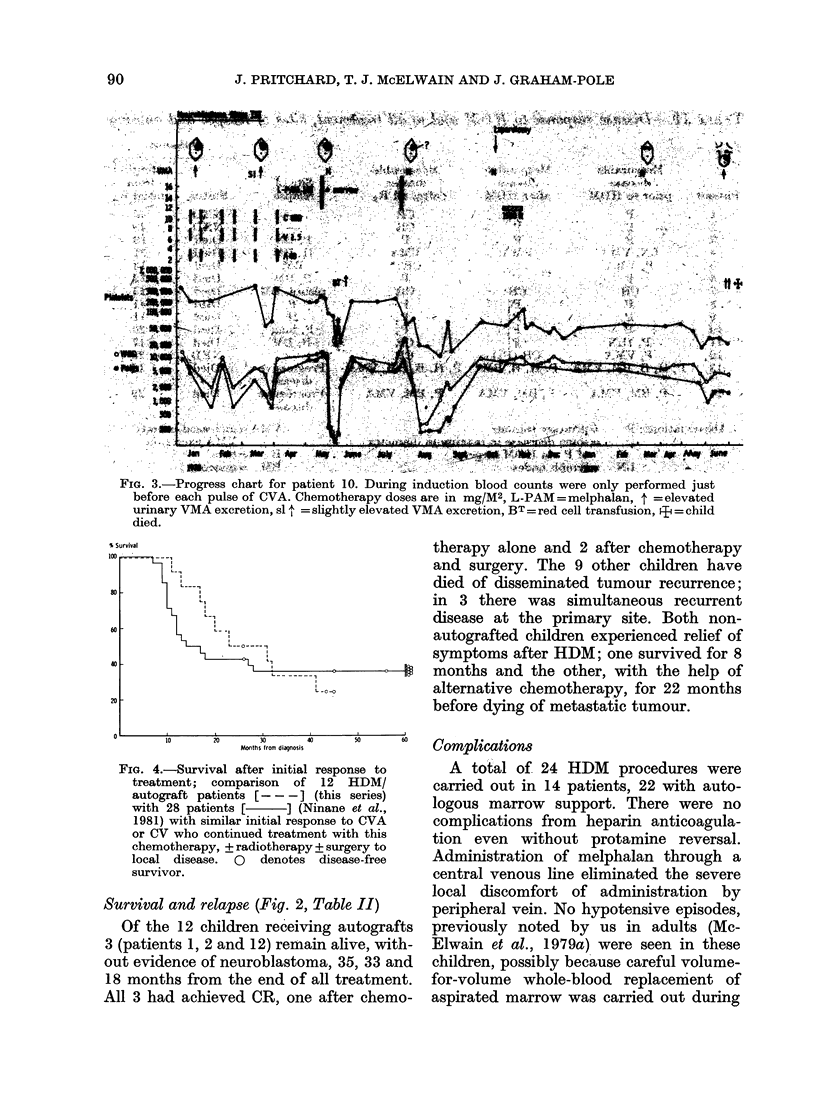

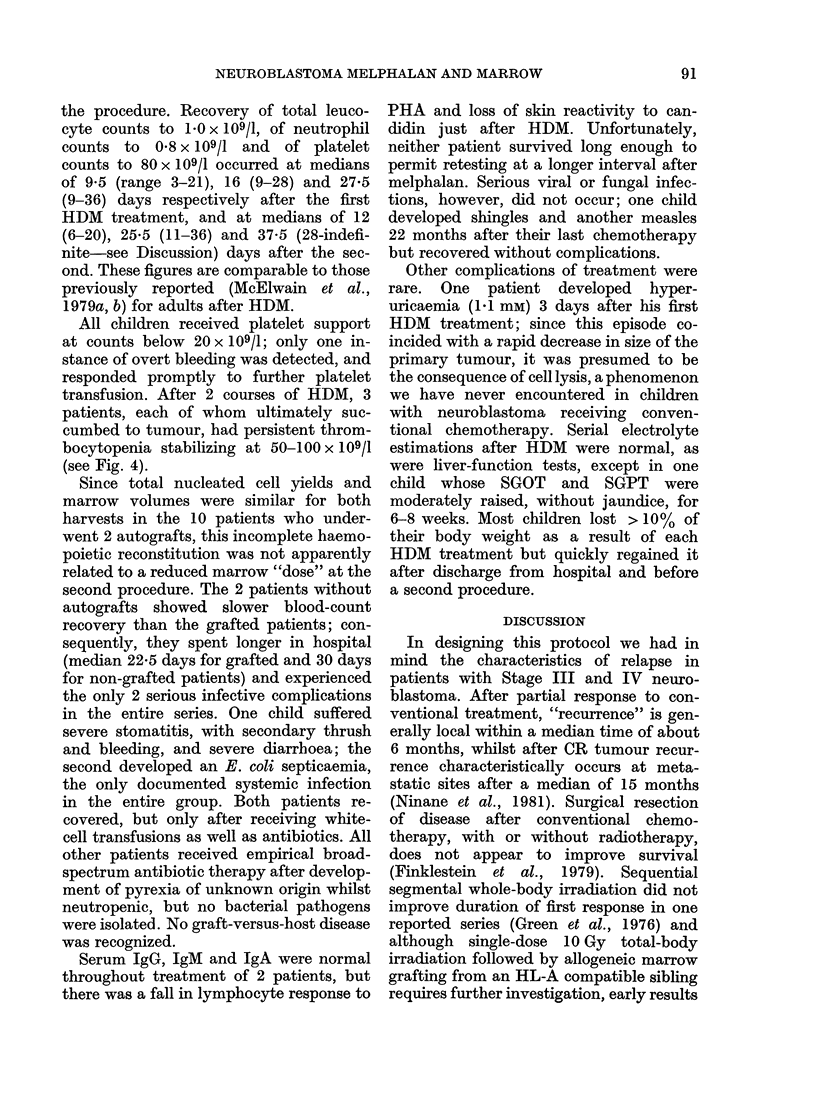

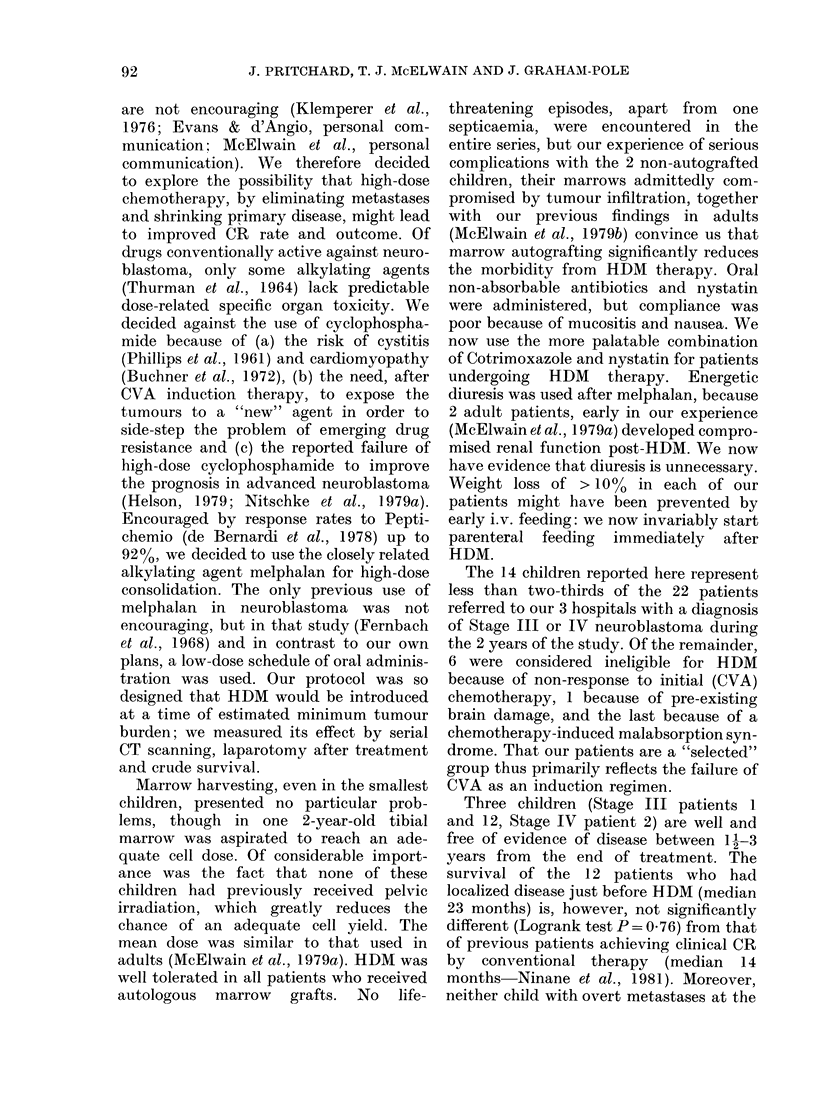

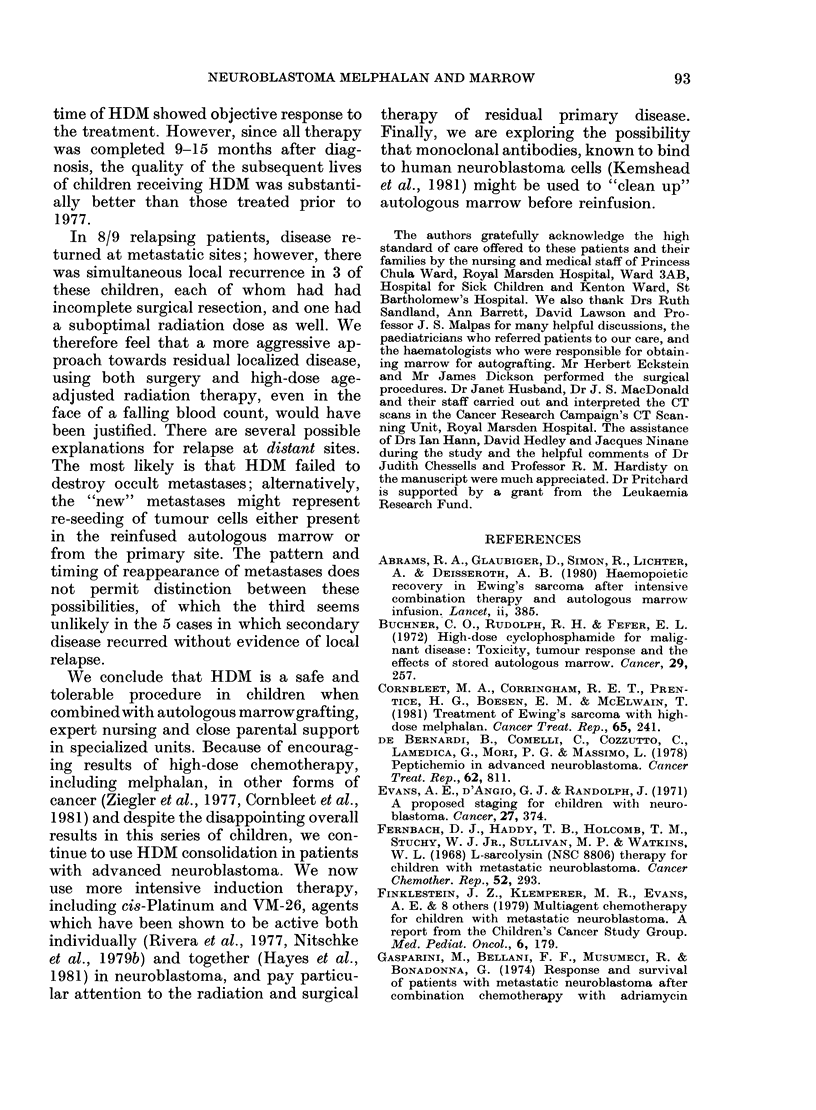

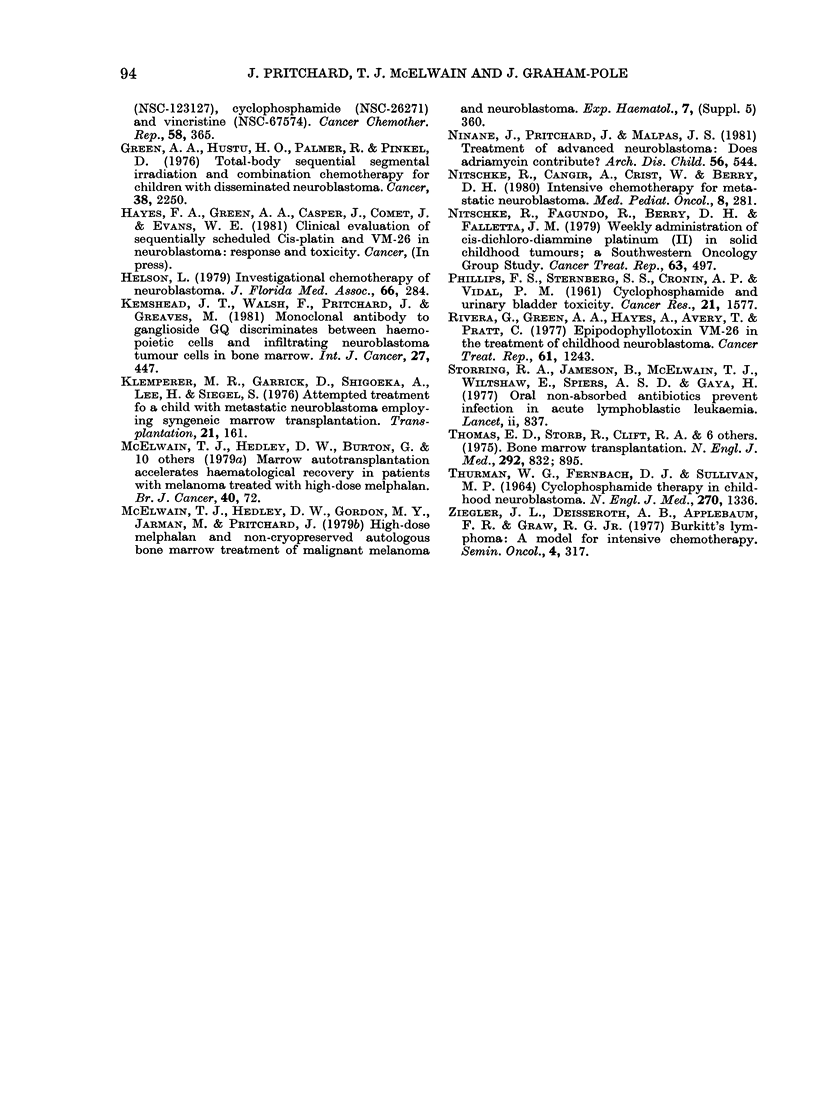


## References

[OCR_00931] Abrams R. A., Glaubiger D., Simon R., Lichter A., Deisseroth A. B. (1980). Haemopoietic recovery in Ewing's sarcoma after intensive combination therapy and autologous marrow infusion.. Lancet.

[OCR_00947] Cornbleet M. A., Corringham R. E., Prentice H. G., Boesen E. M., McElwain T. J. (1981). Treatment of Ewing's sarcoma with high-dose melphalan and autologous bone marrow transplantation.. Cancer Treat Rep.

[OCR_00951] De Bernardi B., Comelli A., Cozzutto C., Lamedica G., Mori P. G., Massimo L. (1978). Peptichemio in advanced neuroblastoma.. Cancer Treat Rep.

[OCR_00957] Evans A. E., D'Angio G. J., Randolph J. (1971). A proposed staging for children with neuroblastoma. Children's cancer study group A.. Cancer.

[OCR_00962] Fernbach D. J., Haddy T. B., Holcomb T. M., Stuckey W. J., Watkins W. L. (1968). L-sarcolysin (NSC-8806) therapy for children with metastatic neuroblastoma.. Cancer Chemother Rep.

[OCR_00969] Finklestein J. Z., Klemperer M. R., Evans A., Bernstein I., Leikin S., McCreadie S., Grosfeld J., Hittle R., Weiner J., Sather H. (1979). Multiagent chemotherapy for children with metastatic neuroblastoma: a report from Childrens Cancer Study Group.. Med Pediatr Oncol.

[OCR_00976] Gasparini M., Bellani F. F., Musumeci R., Bonadonna G. (1974). Response and survival of patients with metastatic neuroblastoma after combination chemotherapy with adriamycin (NSC-123127), cyclophosphamide (NSC-26271), and vincristine (NSC-67574).. Cancer Chemother Rep.

[OCR_00988] Green A. A., Hustu H. O., Palmer R., Pinkel D. (1976). Total-body sequential segmental irradiation and combination chemotherapy for children with disseminated neuroblastoma.. Cancer.

[OCR_01002] Helson L. (1979). Investigational chemotherapy of neuroblastoma.. J Fla Med Assoc.

[OCR_01005] Kemshead J. T., Walsh F., Pritchard J., Greaves M. (1981). Monoclonal antibody to ganglioside GQ discriminates between haemopoietic cells and infiltrating neuroblastoma tumour cells in bone marrow.. Int J Cancer.

[OCR_01013] Klemperer M. R., Ganick D., Shigeoka A., Lee H., Segel G. (1976). Attempted treatment of a child with metastatic neuroblastoma employing syngeneic marrow transplantation.. Transplantation.

[OCR_01027] McElwain T. J., Hedley D. W., Gordon M. Y., Jarman M., Millar J. L., Pritchard J. (1979). High dose melphalan and non-cryopreserved autologous bone marrow treatment of malignant melanoma and neuroblastoma.. Exp Hematol.

[OCR_01036] Ninane J., Pritchard J., Malpas J. S. (1981). Chemotherapy of advanced neuroblastoma: does adriamycin contribute?. Arch Dis Child.

[OCR_01040] Nitschke R., Cangir A., Crist W., Berry D. H. (1980). Intensive chemotherapy for metastatic neuroblastoma: a Southwest Oncology Group study.. Med Pediatr Oncol.

[OCR_01044] Nitschke R., Fagundo R., Berry D. H., Falletta J. M. (1979). Weekly administration of cis-dichlorodiammineplatinum(II) in childhood solid tumors: a Southwest Oncology Group study.. Cancer Treat Rep.

[OCR_01051] PHILIPS F. S., STERNBERG S. S., CRONIN A. P., VIDAL P. M. (1961). Cyclophosphamide and urinary bladder toxicity.. Cancer Res.

[OCR_01055] Rivera G., Green A., Hayes A., Avery T., Pratt C. (1977). Epipodophyllotoxin VM-26 in the treatment of childhood neuroblastoma.. Cancer Treat Rep.

[OCR_01061] Storring R. A., Jameson B., McElwain T. J., Wiltshaw E. (1977). Oral non-absorbed antibiotics prevent infection in acute non-lymphoblastic leukaemia.. Lancet.

[OCR_01073] THURMAN W. G., FERNBACH D. J., SULLIVAN M. P. (1964). CYCLOPHOSPHAMIDE THERAPY IN CHILDHOOD NEUROBLASTOMA.. N Engl J Med.

[OCR_01068] Thomas E. D., Storb R., Clift R. A., Fefer A., Johnson L., Neiman P. E., Lerner K. G., Glucksberg H., Buckner C. D. (1975). Bone-marrow transplantation (second of two parts).. N Engl J Med.

[OCR_01077] Ziegler J. L., Deisseroth A. B., Applebaum F. R., Graw R. G. (1977). Burkitt's lymphoma--a model for intensive chemotherapy.. Semin Oncol.

